# Job Satisfaction and Performance Orientation of Paramedics in German Emergency Medical Services—A Nationwide Survey

**DOI:** 10.3390/ijerph182312459

**Published:** 2021-11-26

**Authors:** Christian Eiche, Torsten Birkholz, Fabian Konrad, Tobias Golditz, Johann Georg Keunecke, Johannes Prottengeier

**Affiliations:** 1 Department of Anesthesiology, University Hospital Erlangen, 91054 Erlangen, Germany; torsten.birkholz@uk-erlangen.de (T.B.); fabian.konrad@uk-erlangen.de (F.K.); tobias.golditz@uk-erlangen.de (T.G.); georg.keunecke@uk-erlangen.de (J.G.K.); johannes.prottengeier@uk-erlangen.de (J.P.); 2 Faculty of Medicine, Friedrich-Alexander University Erlangen-Nuremberg, 91054 Erlangen, Germany

**Keywords:** emergency medical services, EMS, paramedics, labor shortage, shortage of skilled workers, job satisfaction, performance orientation

## Abstract

(1) Background: Shortage of skilled workers is a relevant global health care problem. To remain competitive with other professions, job satisfaction is a critical issue; however, to date, there are no data available on the German EMS. This study aims to perform a statistical analysis of job satisfaction and performance orientation and to identify risk factors for low job satisfaction of paramedics in the German EMS. (2) Methods: Data were collected from 2590 paramedics through a nationwide cross-sectional survey, using the job satisfaction questionnaire by Neuberger and Allerbeck and the performance orientation questionnaire by Hippler and Krüger. Descriptive and regression statistical analysis were performed. (3) Results: The participants scored significantly lower than the reference sample on job satisfaction, with “organization and management” and “payment” being the lowest rated subscales. Around 9% of employees feared losing their jobs. While work attitude toward performance and success enhancement was high, fear of failure was also common. (4) Conclusions: Job satisfaction of paramedics in the German EMS is below that of the reference sample. Discontent with payment and organizational issues is common. Performance orientation is high, but fear of failure is frequent. Current and future efforts that aim at an attractive working environment should reflect on these findings.

## 1. Introduction

Rising numbers of emergency medical service (EMS) missions and a developing shortage of skilled health care workers are global issues [1]. The necessity of emergency medical workers and their contribution to the medical services has been illuminated by the recent COVID-19 pandemic. The public became more interested in the working conditions of paramedics and nurses, as they were battling the virus pandemic at the very forefront. The alarming shortage of these health care workers has displayed one of the weak points of health care systems around the world.

Even before the COVID-19 pandemic, the number of emergency and non-emergency missions together increased by nearly 50% between 1994 and 2013 in the German EMS [2]. Consequently, the number of EMS employees increased by nearly 48% from 2012 to 2018 [3]. However, forecasts indicate a steadily increasing shortage of employees not only in Germany [4], but globally [5,6].

Among paramedics in the US, the annual turnover in employees is almost 10% [7,8]. In Israel, Dopelt and colleagues report turnover rates, among paramedics after two years of training, of 42% and after 10 years of training of over 90% [9]. Recent studies in Germany have shown similar, alarmingly high numbers. A total of 54% of paramedics were considering leaving the EMS within the following year [10], 46% of them were dissatisfied with their job. It has also been observed that paramedics have a relatively lower rate of well-being and a higher rate of depression [10] and burnout [11]. Prehospital emergency medicine is considered as an occupation in which employees are regularly confronted with EMS-specific external stressors, such as critically ill patients, accidents but also physical strain and danger [12]. Internal and external stressors are important determinates for job satisfaction. 

Turnover of employees is not only associated with high workload for employers and torn personal relationships but also with high costs and the loss of knowledge and experience. Retention of new well-trained employees in the EMS is difficult and highly competitive among the agencies [8].

Low job satisfaction can be problematic both in terms of shortage of skilled workers and the health of each employee. In a study by van der Ploeg et al., factors such as lack of social support from colleagues and supervisors as well as poor communication were found to be significant predictors of posttraumatic stress disorder, burnout symptoms, and fatigue [12]. These symptoms again are significantly associated with negative safety outcomes among EMS personnel [10]. A critical association between burnout, well-being, and procedural errors has been consistently reported in health care studies [13]. Aiken et al. found that hospitals with a positive work environment have more satisfied nurses leading to better quality and safety of care and more satisfied patients. Improving the hospital work setting could be an organizational strategy to improve patient outcomes and to retain qualified staff [14]. Another study demonstrated a positive relationship between job satisfaction and job performance among nurses [15]. It is conceivable that this relationship also applies to the EMS. This elucidates the importance of job satisfaction, not only for personal reasons, but also in terms of work safety and quality. However, as shown by Streud et al., low job satisfaction after a one year follow-up was mainly predicted by universal occupational stressors, such as a lack of support from leaders and coworkers, not by EMS-specific factors [16].

Beside the external factors—such as the swelling number of emergency missions and a growing share of elderly people in the population—internal stressors, such as structural changes in the EMS system itself, with rising responsibility for the paramedics and increasing cost pressure for the EMS system in general, are putting the EMS system in Germany under steadily growing pressure.

In 2017, the German EMS employed approximately 62,000 people, of whom, 24% were women [17]. It is an emergency physician-based EMS system. In severe cases, the paramedics assist the physician in charge at the scene, while the paramedics handle low-acuity cases by themselves.

In the last few years, a significant change has taken place in the German EMS. It has been restructured, and the new profession of “Notfallsanitäter” has been introduced. At this moment there are three categories of paramedics. The path to the new profession of “Notfallsanitäter” comprises a three year vocational training. “Notfallsanitäter” are legally allowed to perform certain tasks at their own discretion that were previously performed by physicians only, and they usually act as the team leader of an emergency ambulance crew. The previous profession of “Rettungsassistent,” which required a two year training period, can still be considered as the team leader of an ambulance crew, but in a few years, all “Rettungsassistent” will be required to complete additional training if they want to remain in this position. The third category of “Rettungssanitäter” requires only 13 weeks of training and involves subordinate duties and responsibilities.

This structural change means an increase in responsibility for paramedics, who will now make decisions about how to treat patients without the backup of an emergency physician. At present, it is unclear how this change will affect the meta-levels of paramedics’ work. There are no data available addressing issues such as performance orientation, career orientation, performance-enhancing work attitudes, or fear of failure. In our opinion, the high level of quality of medical procedures, as currently performed by physicians, must be maintained—without overwhelming paramedics with their new responsibilities. To date, there is also no detailed information available that could help in the analysis of the different aspects of job satisfaction and in the possible explanations for the high rate of dissatisfaction among employees in the German EMS. This study aims to perform a statistical analysis of job satisfaction and performance orientation and to identify risk factors for low job satisfaction of paramedics in the German EMS.

## 2. Materials and Methods

In autumn 2017, a nationwide, German, cross-sectional questionnaire survey was conducted to collect sociodemographic data on ambulance workers and to identify indicators of job satisfaction and performance orientation. The study was widely advertised through information letters, posters, and flyers that were sent to all German EMS stations and paramedic academies. Announcements were printed in all major German EMS journals. Relief organizations, labor unions, and the German Association of Paramedics (Bundesverband Rettungsdienst) have supported our study through their online communications.

The survey was conducted as an online questionnaire provided via the SoSci-Survey platform. Participation was voluntary and anonymous.

Questionnaires with more than 20% missing answers were excluded from the analysis. The emergency physicians were not included in the survey because of their different working conditions.

The University of Erlangen-Nuremberg’s research ethics committee approved of our study by formal decision (Decision Number 172-17B). The need for formal consent from the participants was waived by the ethics committee, and voluntary participation served as a surrogate for consent.

To collect the participants’ sociodemographic data, questions about age, gender, height, and weight were included. Height and weight were recorded, because work in the EMS is often associated with physical strain. Information about professional experience was also collected, including the highest level of professional training, number of years spent working in EMS, and an estimate of the total number of emergency missions handled to date.

Questionnaires developed by previous studies were used to identify indicators for job satisfaction and performance orientation: the job satisfaction questionnaire by Neuberger and Allerbeck [18] and the performance orientation questionnaire by Hippler and Krüger [19].

The job satisfaction questionnaire by Neuberger and Allerbeck consists of 7 subscales. Each subscale contains several questions to investigate on every aspect of the subscale. In total, 79 items are included. Every item is rated on a scale from 1 = “yes” to 4 = “no.” Since “yes” is positive for some items and negative for others, some items have to be reversed. The mean of the added values indicates the level of satisfaction from 1 = low satisfaction to 4 = high satisfaction. Table 1 shows an overview of the subscales.

Since questions of the different subscales vary in their complexity of subjective assessment, a cumulative question at the end of each subscale “All in all, how satisfied are you with…”—was asked to allow direct comparison of subscales. This question was presented using Kunin faces, ranging from 1 = very dissatisfied (face with a downturned mouth) to 7 = very satisfied (laughing face). Other collective questions, such as “All in all, how satisfied are you with your job?” and “All in all, how satisfied are you with your life?”, were also rated on a 7 item Kunin face scale.

Additionally, the questionnaire addresses the arrangement of working hours and asks about fear of job loss. The participants were able to indicate the degree of their approval to the questions “The danger of losing my job is high” and “I am satisfied with the arrangement of my working hours” with “yes,” “rather yes,” “rather no,” and “no.”

To relate job satisfaction of EMS employees with other professions, the current data was compared to a standard reference sample, provided by Neuberger, which has been made up of more than 2000 employees of various professions [20].

The performance orientation questionnaire by Hippler and Krüger consists of 26 items organized into three subscales: “career orientation,” “performance- and success-enhancing work attitude,” and “fear of failure.” Each item can be rated from 1 (“I do not agree at all”) to 7 (“I completely agree”). At the end, the mean of all items is calculated. The career orientation subscale mainly includes aspects of future orientation, resilience, assertiveness, social competition, and level of ambition. The performance- and success-enhancing work attitude subscale primarily consists of aspects of persistence, professional task completion, and assertiveness. The last subscale, fear of failure, consists of negative feelings, such as fear of embarrassment, worry about important events, and preference for tasks with a foreseeable end.

### Statistical Analysis

The data we gathered is available via a persistent digital object identifier (DOI), linking to the dataset stored in the Zenodo data repository. Statistical analysis was performed using SPSS Statistics 24.0.0.0 (IBM Corp. Armonk, NY, USA), and statistical significance was defined as *p* < 0.05. Values are presented as means with standard deviations and medians with interquartile ranges, where appropriate.

We used descriptive statistical analysis to describe the underlying study population in terms of age, gender distribution, BMI, level of training, and work experience.

The aim of this study was to investigate the job satisfaction of paramedics in German EMS. In a first step, the results were put in comparison to a general representative sample of German employees. In order to elucidate the differences of the representative sample and the mean values of the paramedics group, a one-sample *t*-test was used. The Wilcoxon test was used to compare items in the questionnaire that used rating scales.

Since there are significant differences between the representative sample and the paramedics group, we investigated the results of the paramedics group more closely in a second step. To identify causes of low job satisfaction, we divided the paramedics in two groups. Group 1, with low job satisfaction, who chose a Kunin face with a downturned mouth (1–3), were compared with those with high job satisfaction, who chose a Kunin face with an upturned mouth (5–7). Afterwards, a binary regression analysis with a stepwise selection of predictor variables was carried out. Job satisfaction was thereby used as the dependent variable, whereas the paramedics’ personal characteristics were used as independent variables.

## 3. Results

A total of 2731 employees completed the questionnaire. Due to the described mode of questionnaire dissemination, a distinct response rate could not be calculated.

In sum, data from 2590 paramedics could be analyzed. The sample size for an analyzed items may vary from the total sample size, due to incomplete answers.

Table 2 shows an overview of the basic sociodemographic data.

### 3.1. Job Satisfaction

In the first step of our analysis, the group of paramedics were put in comparison to the representative sample to investigate the difference of EMS employees from the general population. In this comparison, statistically significant differences were found for all subscales of job satisfaction between the cohort of paramedics and the representative sample provided by Neuberger [20], which should mirror the average population.

Each subscale was rated lower by the paramedics. The categories of “organization and management” and “my payment” showed the biggest differences when compared with the reference group’s responses; whereas the categories of “my development” and “my activity” showed the smallest differences. Table 3 gives an overview of the different subscales, both in the present data set and in the representative sample.

**Figure 1:**
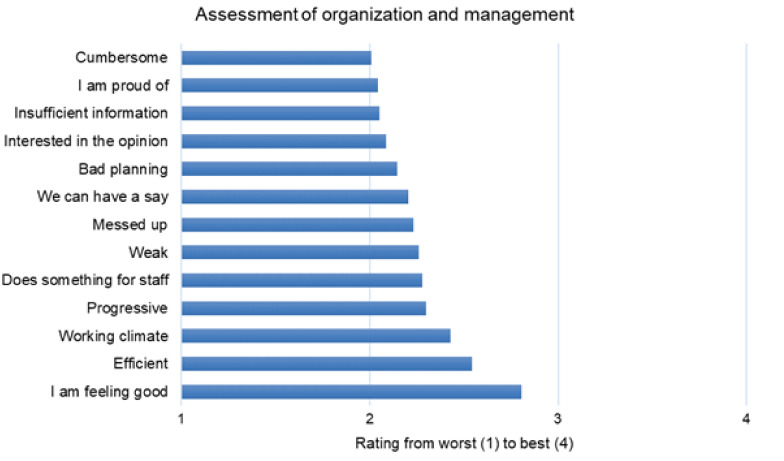
Detailed answers for the subscale “Organization and Management”.

**Figure 2:**
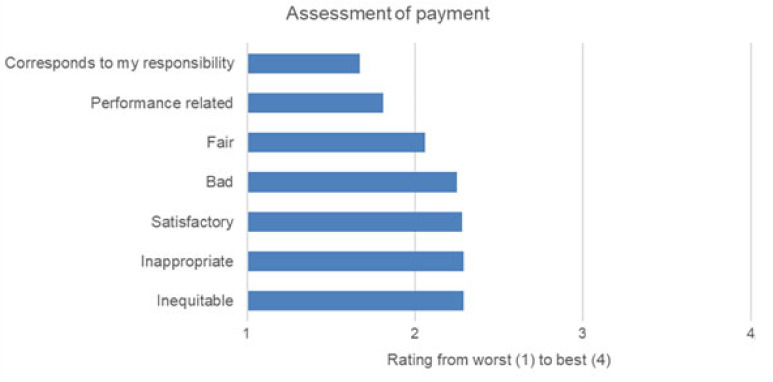
Detailed answers for the subscale “Payment”.

The two worst-rated categories, “organization and management” and “payment,” were examined more closely at the single item level. Figure 1 and Figure 2 show the results for each question. For a better overview in the figure, we used the scale “best” to “worst,” instead of “yes” to “no,” as it was done in the original questionnaire, since some questions were formulated positively, and some were framed negatively and had to be inverted.

For the category “organization and management,” the participants indicated that higher management was cumbersome, were uninterested in the opinions of paramedics, and only provided insufficient organizational information, and that employees were not proud of their organization (Figure 1). For the category “payment,” the detailed answers showed that most of the paramedics did not think their pay corresponded to their responsibilities, and that pay was not related to performance (Figure 2).

In a second step, every subscale was rated in a summarizing question—“All in all, how satisfied are you with …”—was rated on a scale of 7 Kunin faces, ranging from 1 (lowest satisfaction) to 7 (highest satisfaction).

**Figure 3:**
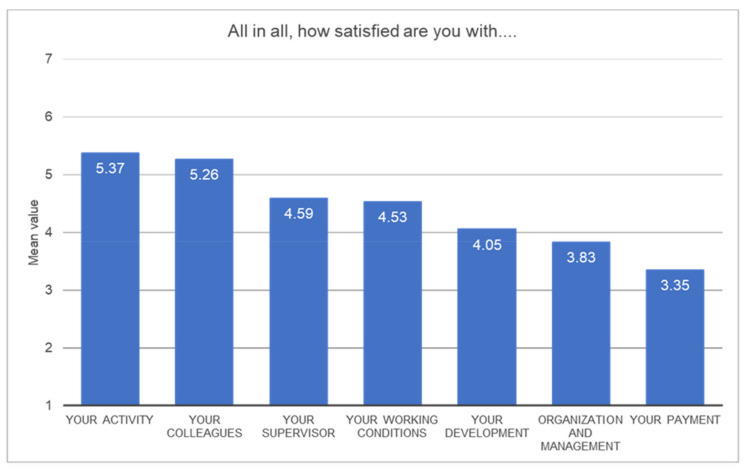
Mean values of different job satisfaction categories (1 = low satisfaction–7 = high satisfaction).

The data shows that the subscales “my activity” and “my colleagues” were rated the highest. Again, the worst-rated categories were “organization and management” and “payment.” Table 4 shows an overview of the different categories. Figure 3 presents a graphical overview.

In terms of the summary question—“All in all, how satisfied are you overall with your job?”—16.5% of the participants chose a negative Kunin face (downturned angle of mouth), and 65% picked a positive Kunin face (upturned angle of mouth). For the question “All in all, how satisfied are you overall with your life?”, 20.4% selected a negative Kunin face, and 63.4% chose a positive Kunin face. Job and life satisfaction showed no significant difference using Wilcoxon’s test (Z = −0.54; *p* = 0.59).

Most participants were not afraid of losing their job. The question “The danger of losing my job is high” was answered with “no” or “not very much” by more than 90% of the participants. Satisfaction with the arrangement of working hours widely varied between the participants. Approximately half were more satisfied, and the rest were more dissatisfied. Table 5 gives an overview.

In a third step, a binary logistic regression analysis was conducted to identify factors that influenced overall work satisfaction. The basic sociodemographic data of paramedics who chose a Kunin face with a downturned mouth (1–3) were compared to those who chose a Kunin face with an upturned mouth (5–7). By this analysis, we were able to identify factors that were associated with higher job satisfaction, as follows:

0 to 5 years of service (OR = 1.58, *p* < 0.01); ages between 18 and 30 years (OR = 1.98, *p* < 0.01), and between 31 and 40 years (OR = 2.26, *p* < 0.01); being “Notfallsanitäter in training” (OR = 3.09, *p* = 0.03) resulted in a higher probability of reporting high job satisfaction; being qualified as “Rettungsassistent” resulted in a probability of reporting low job satisfaction (OR = 0.69, *p* < 0.01) (Table 6). The other independent variables showed no significant association.

The complete model was significant (chi-squared (12) = 102.10, *p* < 0.01). Nagelkerke’s R2-value was 0.075. Table 6 shows more detailed information on the regression analysis.

### 3.2. Performance Orientation

The questionnaire by Hippler and Krueger was used to elicit data on performance orientation. Each subscale has a possible value from 1 (low) to 7 (high) points.

The paramedics reported a mean career orientation value of 4.34 points. Only a few paramedics had a very strong or a very weak career orientation.

Compared with career orientation, performance- and success-enhancing work attitudes showed higher values. The mean was 5.53, and most of the paramedics had a high interest in carrying out the tasks assigned to them professionally.

The mean for the subscale “fear of failure” was 4.55. For this item, higher scores indicate a greater fear of failure.

## 4. Discussion

The shortage of skilled workers is a relevant problem in German health care, and it is predicted to worsen in the coming years. For a health discipline to remain competitive in comparison to other occupations, job satisfaction is a critical factor. To date, there is no nationwide data on job satisfaction in paramedics. In our study, we included a dataset with more than 2500 employees, and found that low job satisfaction is a pertinent issue in the German EMS.

### 4.1. Job Satisfaction

The question “All in all, how satisfied are you with your job?” was answered with a negative Kunin face by 16.5% of participants. This was a lower rate of dissatisfaction among paramedics than was shown by Baier et al., but it still indicates a need to be concerned, given the forecast of skilled worker shortages [10]. To remain competitive with other occupations, it should be a goal for the EMS sector to achieve results at least similar to those of other professions, as provided by Neuberger [20]. Cydulka reported that 89% of paramedics described their job as stressful and were mentally worn-out and exhausted [21]. Years of service as EMS paramedic correlates with work exhaustion [22], which could be an explanation for the high turnover of employees in EMS.

On a positive note, core EMS tasks themselves achieved the highest ratings compared with all other areas of job satisfaction. There was no significant difference to the representative sample. Furthermore, the worst-rated areas are amenable, in principle, to change and improvement.

In numerous previous studies personal relations to coworkers played a key role in job satisfaction among EMS paramedics [9,21,22]. Strong camaraderie can be a valuable asset in generating a healthy work environment [23]. Interaction with coworkers has been rated the highest in our survey. Personal interactions and the atmosphere in the team are known as protective factors through studies on nurses [24] and physicians [25].

Aspects of “organization and management” and “salary” scored lowest in our study population. The discontent with the salary agrees with previous results from studies among American [22,23] and Israelian paramedics [9]. Since these were significant determinates for job dissatisfaction, the responses to individual items in these subscales were examined more closely.

In the category “organization and management,” three out of the six worst-scoring questions were related to management–employee communication. The participants’ responses indicate that employees think they do not receive sufficient information, and that management is not interested in their opinions. These areas might be a starting point for improving employee satisfaction. Interestingly, these findings have not been reported in previous, international studies [9,22,23].

The difficulties in management–employee communication could arise due to the situation in Germany, in which several independent relief organizations are responsible for the emergency services across the country. It might be challenging to get all employees to the same level of knowledge when there are several different organizations in one city.

Regular informational letters and the opportunity for employees to submit suggestions for improvement to the management could be easy ways to improve job satisfaction. The EMS management could look for suggestions in other occupational fields, in which comprehensive job satisfaction strategies have been successfully implemented.

Interestingly, the participants’ direct supervisors received much better ratings than the superimposed levels of organization and management. The reasons for this finding remain unclear, but it is possible that direct supervisors have a better understanding of their employees’ needs, because they are mostly paramedics themselves, while higher-level management consists of physicians, politicians, and economists.

The worst category of job satisfaction was the participants’ pay. An analysis of individual questions related to payment indicated that the paramedics did not think their salary corresponded to their responsibilities. These findings go along with results from American paramedics, who were least satisfied with payment and the possibilities of advancement [23]. Dopelt et al. report similar results from Israel [9]. Israelian paramedics reported discontent with low wages and the lack of career opportunities. They also reported a growing influence of work on their private lives, compromising family live and marriage [9].

This finding becomes even more worrisome, given that in the future, additional medical measures will be performed by paramedics rather than by physicians. Decision-makers should carefully weigh whether this extension of responsibilities should be compensated for in monetary terms.

The item “The danger of losing my job is high” was answered as “no” or “not very much” by more than 90% of the participants. This finding, which appears positive at first glance, might also be indicative that employers would avoid losing employees at all costs because of an impending shortage of skilled workers.

Another aspect of improving job satisfaction could be a more flexible scheduling of work shifts, as only 23.2% of the participants answered the question “I am satisfied with the arrangement of my working hours” with “yes”, while 17.6% answered with “no”. The reasons for this wide range of satisfaction levels remain unclear, but if further research indicates that it could depend on different duty rosters and not on personal factors, then there could be a potential for optimization.

#### 4.1.1. Influencing Factors

In a binary regression analysis, we were able to identify some risk factors for low job satisfaction. Although these factors were mathematically significant, we will not discuss them further because Nagelkerke’s R2-value was only 0.075, which indicates a poor model fit. Other influencing factors on job satisfaction must be present that were not part of our questionnaire. It is possible that family and social background play an important role. Further investigations into these and other causes of low job satisfaction are needed at this point.

#### 4.1.2. Performance Orientation

Finally, we found a strong performance- and success-enhancing work attitude in paramedics. This indicates that most paramedics want to adequately perform the tasks assigned to them professionally. One of the reasons for low-skilled paramedics to consider leaving the EMS is the motivation for further education [22]. Especially regarding the planned extension of medical measures, these are reassuring results and a positive sign for future actions. However, fear of failure must be taken very seriously so as not to overwhelm paramedics. Overall, it is important to create a working environment in which performance orientation of paramedics is encouraged without overstraining employees. There are protective factors to ensure an enduring healthy work attitude. Paramedics who are able to maintain good physical health seemed to be less likely to leave the EMS [26]. However, results from Studnek et al. [27] described almost 25% of paramedics as obese.

In particular, sound training and regular evaluations of both performance levels and paramedics’ feelings could play an important role. Hopefully, a sustainable working environment can be created in this way.

Additionally, since all medical professions alike are threatened by a shortage of skilled labor, we recommend performing more studies of this kind on other medical professions.

#### 4.1.3. Limitations

Naturally, our findings come with limitations. Despite the study’s extensive distribution across the entire German EMS, a relevant selection bias of participants cannot be ruled out. It remains uncertain what motivated paramedics to participate in this study, and whether the willingness to participate is correlated with the variables investigated in this study.

Secondly, in this study, the subjective ratings gathered from employees were not paired with corresponding assessments from the employers. It is conceivable that views on various items might be different depending on the perspective people may have regarding the employment contract.

Moreover, it will be difficult to compare the current findings with data from other countries, as divergent cultural and economic backgrounds may have a major impact on employees’ ratings of their job and on the effort they are putting in.

## 5. Conclusions

An assessment of the German paramedics’ jobs reveals that they are most satisfied with the nature of their work and with their immediate colleagues. They have little fear of losing their employment. However, they are least satisfied with their organization’s management and their pay. Efforts to improve job satisfaction could work on both top-to-bottom and bottom-to-top communication patterns as “low-hanging fruits” of success. The high-performance orientation of paramedics is reassuring, considering the vital responsibilities of their profession. However, future extensions of their duties should reflect on the fear of failure as a common trait in their cohort. Finally, interventions and future developments in the EMS profession should be evaluated with regard to their effect on job satisfaction and performance orientation, by repeating our assessment at regular intervals.

## Figures and Tables

**Table 1:** Subscales of the job satisfaction questionnaire by Neuberger and Allerbeck.

Subscale	Description of the Subscale
My colleagues	This refers to the colleagues with whom you work directly and have work-related contact.
My supervisor	This refers to your immediate superior (who is one step higher than you are and who gives you instructions and directs you).
My activity	This refers to the content and the nature of your job.
My working conditions	This refers to the conditions under which you work (e.g., tools, machinery, workspace, environment, noise, temperature, etc.).
Organization and management	This category refers to how you see the company as a whole; how the cooperation between the divisions and departments works; how you assess regulations, planning, and information; and the “top” management.
Development	This refers to your personal progress (your current and future opportunities for promotion, further education, and the assumption of tasks with greater responsibility).
My payment	This refers to the payment you receive for your work (including bonuses, surcharges, 13th salary, etc.)

**Table 2:** Basic sociodemographic data of the participating paramedics.

		Number	Percent
Age (years)	18–30	1152	44.5%
	31–40	722	27.9%
	41–50	504	19.5%
	51–70	208	8.0%
Gender	Male	2079	80.4%
	Female	508	19.6%
Weight	Underweight	14	0.5%
	Normal weight	1018	39.4%
	Pre-obese	978	37.9%
	Obese	573	22.2%
Highest level of professional training (years of training)	Notfallsanitäter in training	98	3.8%
	Rettungssanitäter (13 weeks)	483	18.6%
	Rettungsassistent (2 years)	1177	45.4%
	Notfallsanitäter (3 years)	832	32.1%
Years of service	0–5	899	34.8%
	6–15	851	32.9%
	≥16	833	32.2%

**Table 3:** Mean job satisfaction values compared to the representative sample (range: 1 = low satisfaction to 4 = high satisfaction).

	*n*	Mean for Paramedics	Representative Sample	Difference *	Significance
My activity	2506	3.11	3.19	−0.08	*p* < 0.01
My colleagues	2531	2.99	3.24	−0.25	*p* < 0.01
My supervisor	2538	2.83	3.03	−0.2	*p* < 0.01
My development	2510	2.56	2.63	−0.07	*p* < 0.01
My working conditions	2531	2.47	2.75	−0.28	*p* < 0.01
Organization and management	2485	2.26	2.84	−0.58	*p* < 0.01
My payment	2540	2.09	2.73	−0.64	*p* < 0.01

* the difference is negative when satisfaction is worse in paramedics.

**Table 4:** Mean values of different job satisfaction categories and 95% confidence intervals.

“All in All, How Satisfied Are You with…:”	*n*	Mean	95% CI <	95% CI >
…your supervisor	2583	4.59	4.53	4.66
…your colleagues	2582	5.26	5.22	5.31
…your activity	2585	5.37	5.33	5.42
…your working conditions	2583	4.53	4.48	4.58
…organization and management	2579	3.83	3.77	3.89
…your development	2579	4.05	3.98	4.11
…your payment	2587	3.35	3.29	3.42

CI: confidence intervals.

**Table 5:** Results for the fear of losing the job and arrangements of working hours.

	The Danger of Losing My Job Is High			I Am Satisfied with the Arrangement of My Working Hours		
	*n*	Percent	Total Percent	*n*	Percent	Total Percent
No	1703	65.8	67.3	600	23.2	25.3
Not very	594	22.9	90.8	552	21.3	48.5
Somewhat	129	5.0	95.9	764	29.5	80.7
Yes	105	4.1	100	457	17.6	100
Missing	59			217		

**Table 6:** Factors with a significant influence on job satisfaction (odds ratio for high satisfaction).

	S.E	*p*	OR	95% C.I. for OR	
				**Lower**	**Upper**
Qualified as Notfallsanitäter in training	0.53	0.03	3.085	1.10	8.64
Qualified as Rettungsassistent	0.11	<0.01	0.691	0.55	0.86
0–5 years of service	0.16	<0.01	1.588	1.15	2.19
Age 18–30	0.16	<0.01	1.977	1.44	2.72
Age 31–40	0.15	<0.01	2.263	1.71	3.00
Constant	0.11	<0.01	2.404		

S.E: standard error; OR: odds ratio; CI: confidence intervals.

## Data Availability

The complete questionnaire with all original questions is available at the Zenodo data repository: http://doi.org/10.5281/zenodo.3239112; The dataset generated and analysed during the current study is available at the Zenodo data repository: https://doi.org/10.5281/zenodo.3246911.
